# Inflammation and Vitamin D Receptor Polymorphism: Impact on All-Cause and Cardiovascular Mortality in Mexican Women on Dialysis

**DOI:** 10.3390/biomedicines12091990

**Published:** 2024-09-02

**Authors:** Marcela Avila, Carmen Mora, Ma del Carmen Prado-Uribe, Alfonso Cueto-Manzano, Abdul Rashid Qureshi, Bengt Lindholm, Alma Sofía Bernal Amador, Ramón Paniagua

**Affiliations:** 1 Unidad de Investigación Médica en Enfermedaes Nefrológicas, Hospital de Especialidades, CMN SXXI, Instituto Mexicano del Seguro Social, Av. Cuauhtémoc 330, Col. Doctores, Mexico City 06720, Mexico; 2 Unidad de Investigación Médica en Enfermedades Renales, Hospital de Especialidades, CMNO, Instituto Mexicano del Seguro Social, Guadalajara 44320, Mexico; 3 Renal Medicine and Baxter Novum, Department of Clinical Science, Intervention and Technology, Karolinska Institutet, Karolinska University Hospital, Huddinge, 141 86 Stockholm, Sweden

**Keywords:** CKD, dialysis, diabetes, hemodialysis, all-cause mortality, cardiovascular mortality, mineral and bone disorder, inflammation, vitamin D receptor polymorphism

## Abstract

Mineral bone disease (MBD) is common in dialysis patients. Genetics and the hormonal environment influence the clinical picture and outcomes of women. This study aimed to determine how these factors affect mortality. In 234 female dialysis patients on Continuous Ambulatory (48%) or Automated (29%) Peritoneal Dialysis or Hemodialysis (23%), MBD biochemical variables, as well as bone density and genetic Bsm1 polymorphism of vitamin D receptor (VDR) were performed at baseline. The cohort was followed-up by 17 (IQ range 15–31) months. According to VDR polymorphism, the distribution of patients was bb: 64% and BB+Bb: 36%. Fifty-five patients died from all-cause mortality; the hs-C-reactive protein level was the most significant risk in multivariate Cox analysis. Nineteen died from cardiovascular mortality. None of the variables were significant for cardiovascular mortality. Patients with bb plus inflammation had the highest risk in the analysis; the significance persisted after adjustment for age, diabetes, and parathyroid hormone levels HR 2.33 (95% CI, 1.01–8.33) and after further adjustment for time on dialysis, albumin, and Osteoprotegerin levels HR 3.49 (95% CI, 1.20–10.9). The presence of the bb genotype from VDR and inflammation had the highest risk of death from all-cause mortality in females on CAPD, APD, and HD patient.

## 1. Introduction

Mineral and bone disorders are common in patients with chronic kidney disease and are associated with bone mass loss, extra-skeletal calcifications (CKD-MBD), decreased quality of life, increased morbidity, all-cause mortality, and cardiovascular mortality [1,2].

Previous studies have shown markers of inflammation (such as low serum albumin, interleukin 6 (IL-6), C-reactive protein (CRP), and bone and mineral metabolism (such as hyperphosphatemia, hypercalcemia, increased CaxPO_4_ product, secondary hyperparathyroidism, high levels of FGF23, and Klotho) independently or in combination predict adverse outcomes [3,4,5,6], significantly those linked to cardiovascular comorbidity and mortality in end-stage renal disease (ESRD) patients [7,8,9,10].

Serum Vitamin D concentration has been associated with bone mass, insulin sensitivity, BMI, and immune response [11]. Vitamin D acts after binding to its specific receptor (VDR). The polymorphism in Bsm1 is located in the 12q13-14 region, intron 8 (rs1544410); it is focused on G/A change. The mechanism of this polymorphism in VDR gene protein function remains unclear. However, epigenetic factors such as methylation regulate VDR expression in tuberculosis patients, and non-coding RNAs affect the levels and function of VDR [12,13]. Other factors, such as ethnicity, influence the production and stability of the mRNA of the VDR [14]. Selective long chain polyunsaturated fatty acids (PUFAs) bind to the ligand-binding domain of the VDR and lead to transcriptional activation [15].

In this context, Bsm1 has been associated with loss of bone mineral density in healthy and postmenopausal women and women with CKD [16,17]; this suggests estrogen’s participation in the receptor’s functioning. Women on dialysis are at greater risk for bone loss and fracture than men because of decreased estrogen levels and early menopause [18,19].

VDR polymorphism has also been associated with diabetic nephropathy [20], obesity [21], and inflammation [22]. Interestingly, these factors are linked to morbidity and mortality in CKD patients. These similarities allow us to hypothesize that the VDR polymorphism influences the clinical outcomes of patients with CKD. Based on the previous information, the objective of the present study was to determine the association between VDR gene polymorphism, inflammation, and markers of mineral bone disease with the risk of all-cause and cardiovascular mortality in women on dialysis.

## 2. Materials and Methods

### 2.1. Study Populations

A prospective cohort study was performed on 246 prevalent female dialysis patients recruited from five hospitals in Mexico City with chronic dialysis programs belonging to the Instituto Mexicano Del Seguro Social. All patients gave written informed consent, and their confidentiality and anonymity were protected. Patients were agreed to give a blood sample for biochemical tests and obtain their DNA to exclusively determine the bb, Bb, and BB alleles of the vitamin D3 receptor.

All patients were clinically stable and free from infections or acute complications one month before the investigation. We excluded patients with Hepatitis B and HIV seropositive, cancer, immunosuppressive therapy, parathyroidectomy, and steroid therapy. The causes of ESRD were diabetic nephropathy in 66 patients (27%), arterial hypertension in 31 patients (13%), polycystic kidney disease in 20 patients (8%), glomerulonephritis in 14 patients (6%), lupus in 13 patients (5%), pyelonephritis in 4 patients (2%), obstructive uropathy in 4 patients (2%), and other or unknown causes in 94 patients (37%).

Patients received dialysis treatment with Hemodialysis (HD); n = 57, 23%, Continuous Ambulatory Peritoneal Dialysis (CAPD); n = 118, 48%, or Automated Peritoneal Dialysis (APD); n = 71, 29%. Patients were on HD 4 h, three-times-a-week dialysis schedule with bicarbonate buffered dialysis solutions containing 3.0 mEq/L of calcium (Gambro Renal Products, Tijuana, B. C. México). CAPD patients received four exchanges with 2 L bags with dextrose dialysis solutions containing 3.5 mEq/L of calcium and APD, patients also received conventional therapy with dextrose dialysis solutions containing 3.5 mEq/L of calcium (Dianeal, Baxter, Civac Jiutepec, Mor., Mexico). Demographic and clinical data were recorded for each patient during the investigation.

The patients received medications typical for dialysis patients, such as antihypertensive, control of hyperphosphatemia as calcium-containing phosphorus binder: calcium carbonate (CaCO_3_), at a dose of 2.5 g/day. Calcitriol (1,25-(OH)_2_D_3_) as hyperparathyroidism control. It was administered in doses of 0.25 to 0.75 µg/day. Patients were considered to have calcitriol treatment if such treatment had been continued for over a month.

Two-hundred and twenty-nine patients were followed for a median of 17 months (IQR 15–31), and 12 patients were lost for follow-up. The primary cause of death was obtained from the death certificate, and patient files were recorded.

### 2.2. Procedures

All the patients were called for a clinical review, and their demographic and clinical data were recorded. Gynecological and obstetric backgrounds were compiled (number of children, time of breastfeeding, and amenorrhea) as well as medical history of renal disease (original disease, time and modalities of dialysis, diabetic status, history of fractures, symptoms of bone disease, and use of calcitriol and calcium carbonate), along with factors related to calcium metabolisms, such as ingestion of coffee, soft drinks, alcohol, and tobacco consumption. Amenorrhea was defined as the absence of menstruation exceeding six months. Walking difficulty, fractures, and bone pain were classified as present or absent by interview. In HD patients, the blood sample was taken on a day free of HD in conjunction with the time of the BMD test of the calcaneus (see below). The blood sample was centrifuged, and the plasma was stored at −70 °C until assayed. Baseline values are those used to predict mortality.

### 2.3. Biochemical Measurements

After an overnight fast, a venous blood sample was drawn for biochemical analysis. Albumin (Alb, mg/dL), phosphorus (P; mg/dL), and total calcium (tCa, g/dL) were measured by conventional spectrophotometric assays and high-sensitivity C-reactive protein (hs-CRP, mg/dL) by the Tina-quant immunoturbidimetric assay on a Roche/Hitachi 902 (Tokyo, Japan). Intact parathyroid hormone (iPTH, 1-84), in pg/mL, total pro-collagen type1-amino-terminal propeptide (PINP) in ng/mL, and C-terminal telopeptide-β aspartic acid (β-Cross Laps, or β-CTx) in ng/mL were measured by electrochemiluminescence immunoassay (Roche/Elecsys 1010/2010 Roche, Mannheim, Germany). Osteoprotegerin (OPG; ng/mL) and fetuin (g/L) were determined by ELISA (R&D System Inc., Minneapolis, MS, USA) and Epitope Diagnostic Inc. (San Diego, CA, USA), respectively. The present study defined inflammation as hs-CRP > 0.30 mg/dL.

### 2.4. Bone Mineral Density

The bone mineral density (BMD) of the calcaneus was determined by quantitative ultrasound (QUS) on a Sahara Clinical Bone Sonometer (Hologic, Waltham, MA, USA). The method has been described previously [16] An ultrasound beam passes through the calcaneus. This value was previously correlated with X-ray bone mineral density (BMD) of calcaneus measurements in 2208 Caucasian female subjects [23,24]. Results were expressed in BMD in grams of calcium per square centimeter or in *T*-scores (difference in patient results from the mean results obtained in a young adult population of females) and Z-scores (BMD value about age-matched controls). The equipment was calibrated using a phantom at the beginning of the measurements and during the procedure. The Variation Coefficient % in 1 day was 1.2% and 2% during three days. International values have not been defined for osteoporosis with ultrasound equipment; however, in a meta-analysis [25], the cut-off point for osteopenia was considered to be a *T*-score ≤ −1.0, and for osteoporosis, a *T*-score ≤ −2.5 in the calcaneus of healthy women.

### 2.5. Genotyping

Female DNA was extracted from total blood with the QI Amp DNA Blood Midi Kit from Qiagen, (Valencia, CA, USA). PCR products were digested with restriction enzyme Bsm1 to analyze the VDR alleles bb, Bb, and BB. The conditions of the methodology were described in a previous study from our group [16]. Ninety-four healthy Mexican women served as genetic controls.

### 2.6. Statistical Analysis

Unless otherwise indicated, all variables are expressed as mean ± SD or median (IQR: 25th and 75th quartiles), and percentages. Statistical significance was set at the level of *p* < 0.05. Comparisons between the two groups were assessed with Student’s *t* test and the Square Chi test (χ^2^) test. Spearman’s rank correlation was used to determine correlations between variables. Hardy–Weinberg equilibrium was tested by comparing expected and observed genotype frequencies using the Square Chi test (χ^2^). Cox regression analyses were performed since the assumption of the proportionality of the hazards was met for all covariates. To estimate the all-cause mortality hazard ratios, unadjusted and adjusted by: age, time on dialysis (vintage), DM, and basal serum of Alb, iPTH, OPG, and hs-CRP as predicting variables.

These models selected confounders based on presumed pathophysiological pathways, and all covariates satisfied the proportionality assumption. Since *p* values are not adjusted for multiple tests, they must be considered descriptive. All statistical analyses were performed using the statistical software Stata version 22.2 (Stata Corp., College Station, TX, USA).

## 3. Results

**Figure 1:**
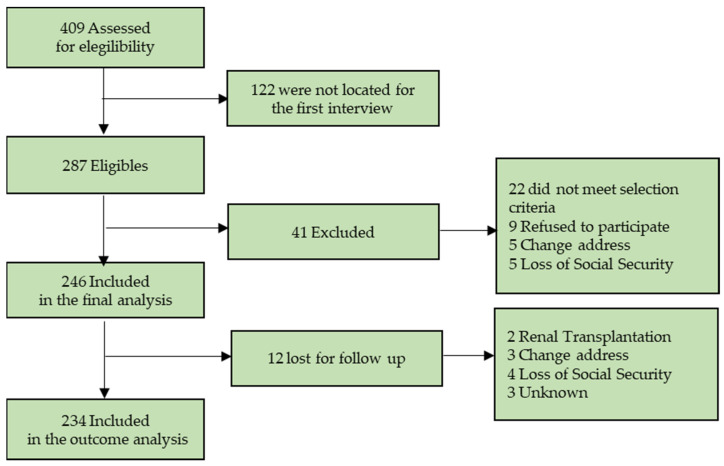
Recruited patient’s flow chart.

Figure 1 shows the recruited patient flow chart: 409 patients were assessed for eligibility at the dialysis centers in Mexico City and surroundings; 122 patients were excluded because they could not be located, and 287 were eligible, of which, 41 were excluded because of several causes: 22 did not meet the selection criteria, mainly because of age, catheter dysfunction, peritonitis, and hospitalizations during the previous month; 9 refused to participate; 5 moved to another city; and 5 lost Social Security coverage. Thus, 246 patients were included in the analysis, 12 were lost for follow-up: 2 had a kidney transplant, 3 changed addresses, 4 lost Social Security, and 3 withdrew from the study for unknown reasons. A total of 234 patients were included in the outcome analysis.

### 3.1. Clinical Characteristics

Table 1 shows baseline clinical and biochemical characteristics of the total population (246 patients) and is classified by Bsm1 polymorphism of VDR: bb (n = 158) and BB+Bb (n = 88). Patients of the bb, Bb, and BB genotypes were 64%, 30%, and 6%, respectively.

For analysis purposes, patients were classified into two groups. One group included patients with bb, and the other group included patients with BB+Bb. Bsm1 polymorphism was in Hardy Weinberg equilibrium in controls and patients. It means that the Genotype distribution was similar in patients and healthy genetic controls.

Patients in the bb group had a lower BMI (24.75 ± 4.09 vs. 26.17 ± 5.6, *p* < 0.02) and a lower serum hs-CRP 0.29 (0.12–0.69) vs. 0.52 (0.21–1.50), *p* < 0.01). Meanwhile, ***T***-score (−1.14 ± 1.1 vs. −1.48 ± 0.97, *p* < 0.023), Z-score (−1.1 (−1.75 to −0.50) vs. −1.30 (−1.3 to −0.9), *p* < 0.01), and Albumin (3.69 ± 0.7 vs. 3.50 ± 0.67, *p* < 0.05) were higher than the BB+Bb group.

Sixty-five patients (26.4%) were treated with vitamin D therapy, Calcitriol. Information was available for 52% of the patients, of whom 85.15% took CaCO_3_.

One hundred thirty-four (55%) patients were classified as inflamed (hs-CRP > 0.3 mg/dL). Hs-CRP had a correlation with BMI (r = 0.213, *p* < 0.01), Fat% (r = 0.208, *p* < 0.001), Glucose (r = 0.149, *p* < 0.029), cCa (r = 0.313, *p* < 0.001), Fetuin (r = −0.204, *p* < 0.0004), extracellular water, (r = 0.213, *p* < 0.006), OPG (r = 0.150, *p* < 0.037).

### 3.2. Outcome and Survival Analysis

There were two hundred and forty-six patients; during the following 17 (IQ range 15–31) months, fifty-five died from all-cause mortality and nineteen (34.5%) from cardiovascular mortality. Twelve were lost from follow-up; the clinical outcome was analyzed in 234 patients. The cause of death was acute myocardial infarction in 5 (9.1%), congestive chronic heart failure in 4 (7.3%), arrhythmia in 5 (9.1%), stroke in 5 (9.1%), infection (except peritonitis) in 8 (14.5%), peritonitis in 1 (1.8%), sudden death in 1 (1.8%), electrolyte disorders in 6 (10.4%), others in 4 (9%), and unknown in 16 (40%) patients who died at home. The patients in the study were subjected to censorship, meaning their data were collected and analyzed from the date of inclusion until one of the following events occurred: renal transplantation, death, or completion of the 36-month follow-up period.

Patients were divided into two groups for survival analysis: survivors (n = 179) and non-survivors (n = 55) (Table 2). Non-survivors were older, had a higher frequency of diabetes, a shorter dialysis history, lower albumin, and also had increased hs-CRP and OPG levels. The frequency of Bsm1 polymorphism was not significantly different among survivors and non-survivors. All other variables were not significant.

The same analysis was performed with cardiovascular mortality, and only the albumin was significant.

Only significant variables from the previous analysis were included in the univariate (one variable at a time) Cox proportional hazard model (Table 3). We found that all-cause mortality was associated with age, diabetes, a low concentration of albumin, and high levels of OPG and hs-CRP; Bsm1 polymorphism was not significant. Treatment with vitamin D tended to be associated with lower mortality but was not significant (HR 0.53 [95% CI 0.24–1.16]; *p* > 0.05). In univariate Cox proportional analysis, age (HR 1.052 (95% CI 1.03–1.103), *p* < 0.032) and DM were the only significant predictors of cardiovascular mortality.

In multivariable (all variables included) Cox proportional hazard model analysis, inflammation (hs-CRP < 0.3 mg/dL) was the only significant predictor for all-cause mortality: HR 1.36 (95% CI 1.11–1.66, *p* < 0.003).

In a multivariate Cox proportional hazard model for cardiovascular mortality, none of the previous variables were significant. This is why they are not shown in Table 3.

It is important to underline that in the crude analysis, considering only inflammation (hs-CRP > 0.3 mg/dL vs. hs-CRP < 0.3 mg/dL), the HR for all-cause mortality was 1.40 (1.18–1.66).

To analyze the possible combined effect of inflammation with other variables on all-cause mortality, the first combination considered was inflammation with polymorphism with no other variables. For this purpose, we made four groups: 1; bb and Non-inflamed, 2; BB+Bb and Non-inflamed, 3; bb and inflamed, 4; BB+Bb and inflamed. The group of patients with bb and inflammation had an increased risk of death HR 2.48 [95% CI 1.08–5.68].

The combined impact of Bsm1 polymorphism and inflammation on all cause-mortality (n = 55) was studied by univariate and multivariate Cox analysis with gradual adjustment for other variables progressively added in subsequent models. These analyses are shown in Table 4.

This risk of bb polymorphism and inflammation persisted at HR 2.33 [95% CI 1.01–5.33] after adjustment for age (per year), Diabetes mellitus, and iPTH (per pg/mL) (Table 4, Model 1). In Model 2, we also adjusted for vintage and albumin (per g/dL), and no significant change occurred. The adjustment for Osteoprotegerin (per unit) and fat body mass percentage increased HR to 3.49 [95%. CI 1.20–10.9, *p* = 0.02]. We included percentage fat in Model 3 to comprehensively explore its potential impact, even though it did not achieve statistical significance in the preliminary analysis.

Inflammation alone had an HR for all-cause mortality of 1.4 and 1.36 in multivariate analysis. Both values were lower than the HR of 2.48 when inflammation was combined with VDR alleles, even after controlling for some other variables, such as those included in Model 1 (2.33) and Model 3 (3.49), shown in Table 4.

**Table 4:** Cox regression analysis for all-cause mortality.

Bsm1	n	Deaths (%)	Model 1	*p*-Value	Model 2	*p*-Value	Model 3	*p*-Value
bb and non-inflamed	8	10.9	1.00		1.00		1.00	
BB+Bb and non-inflamed	30	10.0	0.84 (0.22–3.19)	0.800	0.82 (0.21–3.13)	0.771	1.11 (0.26–4.77)	0.872
bb and inflamed	75	25.3	**2.33 (1.01–5.33)**	**0.042**	**2.38 (1.03–5.50)**	**0.041**	**3.49 (1.20–10.9)**	**0.020**
BB+Bb and inflamed	51	23.5	1.96 (0.78–4.94)	0.151	1.90 (0.75–4.85)	0.17	2.76 (0.88–8.61)	0.072

Death is expressed in %. Hazard ratios, their 95% confidence interval, and significance level are indicated. Model 1 includes Bsm1 genotype plus inflammation, adjusted for age, DM, and iPTH; Model 2 is further adjusted for vintage, serum albumin; Model 3 is further adjusted for OPG, percentage of fat body mass, and Vitamin D therapy.

## 4. Discussion

To our knowledge, this is the first study in female Mexican dialysis patients in which the relationship between Bsm1 polymorphism, inflammation, markers of mineral bone disease, and mortality was analyzed. The current study showed that Bsm1 polymorphism alone does not influence all-cause or cardiovascular mortality in female patients. However, when polymorphism is considered together with inflammation, the risk becomes more significant than inflammation alone, as patients with the bb genotype and inflamed had a higher all-cause mortality risk than those with the BB+Bb genotype and inflamed.

Vitamin D plays a significant role in several metabolic disturbances seen in CKD, 70% to almost 100% of CKD patients showed serum Vitamin D levels below the normal or in the hypovitaminosis limits [26,27], which is why research papers refer to the effect of hypovitaminosis or its changes after supplementation.

Vitamin D and VDR regulate gene expression in mineral homeostasis, skeletal remodeling, renal function, and cardiovascular function. It is also involved in immune regulating cell proliferation, response, and differentiation [28]. For all these actions, Vitamin D requires the activation of its nuclear receptor (VDR) through the endocrine/paracrine and autocrine pathways. As for many other proteins, VDR shows gene polymorphisms; of them, FokI, Bsm1, TaqI, and ApaI have been studied in their association with several diseases. The Bsm1 polymorphism of VDR has been implicated in the BMD [16,17] and iPTH levels [29]. Morrison et al. [30] showed that the BB genotype had a lower BMD than the bb genotype in normal, healthy Caucasian twins.

iPTH concentration was higher than 250 pg/mL in 38% of bb patients and 44% of BB+Bb genotype patients, although iPTH levels were similar in both. Torres et al. [31] found a low PTH in the bb genotype. Notwithstanding, other groups have reported low PTH levels in patients carrying the BB genotype [32,33]. Other factors may have a stronger influence on iPTH than VDR polymorphism, as diabetes, malnutrition, and medication are among them. We did not find differences in iPTH between polymorphisms.

The bb genotype is associated with a higher frequency of inflammation and diabetes) [34]. In México and other developing countries, diabetes is the most common cause of CKD [35]. For more than 40% of the patients on PD in Mexico, the cause of CKD is diabetic nephropathy [36]. For this reason, bb associated with inflammation and diabetes were analyzed.

Our findings suggest that age, diabetes mellitus, and the so-called novel risk factors for cardiovascular disease (such as inflammation, albumin-corrected calcium, and albumin) are associated with survival; this is similar to other studies [37,38], we extended these observations to include levels of serum OPG, as in other studies, OPG was associated with rapid progression of vascular calcification and was a predictor of all-cause mortality in patients on HD and PD [39]. How OPG may operate in vascular pathophysiology has yet to be precisely discovered. However, several clues from clinical studies suggest the role of OPG in vascular calcification.

The frequency of inflammation in patients with PD is estimated at 20% to 70% [40]. The present study showed a high prevalence of inflammation 55%, less than previous studies carried out in our environment (80%) [41]. Inflammation was considered when CRP levels were >3.0 mg/L [42].

In multivariate Cox proportional analysis (Table 3), we found that inflammation was the only factor significantly associated with high mortality when we adjusted for age, vintage, diabetes mellitus, iPTH, Osteoprotegerin, Albumin, and hs-CRP.

The results of the present study showed that Bsm1 polymorphism alone had no significant independent influence on mortality. However, inflamed patients with the bb genotype had a higher risk of mortality than other patient groups (bb without inflammation, BB with or without inflammation). This risk persisted after adjustment for age, diabetes mellitus, and iPTH (Table 4, Model 1); there was no modification after additional adjustment for dialysis vintage and albumin (Model 2). The HR had further increment by adjustment for OPG and percentage of body fat mass (Model 3).

The clinical utility of these results lies in recognizing the prognostic value of hs-CRP (>3.0 mg/L) and bb polymorphism of VDR together as a mortality risk factor. Both must be taken into account to give the best preventive treatment to women on dialysis.

Many factors are involved in the mortality of CKD patients; chronic inflammation is a significant one. In turn, there are many potential sources of inflammation, and hypovitaminosis D, a prevalent finding in CKD, is one of them. Vitamin D levels do not necessarily mean activity; it depends on VDR affinity or functionality.

In a previous study, the effect of vitamin D_3_ supplementation and the influence of Bsm1 in elderly women with vitamin D insufficiency was that the treatment increased the vitamin levels and reduced PTH, CRP, and 1-acid glycoprotein in patients with BB and Bb genotypes but not those with bb [43]. These results are in accordance with our results in the sense that bb patients may have no response to vitamin D_3_ supplements and have a higher risk of all-cause mortality.

Another clinical contribution of our study was that the combination of genomic biomarkers (the VDR gene polymorphism) and clinical factors (inflammation) should be considered for predicting responses to medications related to vitamin D metabolism and mineral bone disease in women on dialysis.

It is important to mention that experimental and clinical Calcimimetic Agents studies have shown to improve hyperparathyroidism and vascular calcification but have not shown to improve cardiovascular mortality, total mortality, or heart failure [44].

Few studies have analyzed the possible association between Bsm1 polymorphism and cardiovascular mortality in dialysis patients. Marco et al. [45] showed that the bb genotype was over-represented among survivors (46%) compared with non-survivors (22%); in contrast, we found that the percentage of bb was similar in survivors (64.8 vs. 65.57%) and non-survivors (35.2 vs. 34.4%). Testa et al. [46] found that the number of BB alleles was associated with the left ventricular mass index in ESRD patients in crude and fully adjusted analyses; however, they did not explore survival. Santoro et al. [47], who found a higher incidence of left ventricular hypertrophy in the subgroup of patients with the BB or Bb genotype. None of these studies analyzed the synergy of polymorphism with other risk markers.

Nevertheless, when we did crude Cox proportional hazards, our results showed no association of serum calcium, phosphorus, calcium-phosphorous product, dialysate calcium concentration, or polymorphism with all-cause and cardiovascular mortality. Contrary, as noted in other studies [1]. Even though the molecular mechanism has not yet been resolved.

The present study has several strengths and limitations that merit discussion. Our study has several strengths, including meticulous phenotyping achieved through detailed anthropometric and laboratory measurements. These measures provided precise information about various aspects of nutritional status, with minimal missing values and biomarkers of mineral metabolism. However, it is imperative to recognize the limitations that accompany our research findings: Firstly, the sample size was small for polymorphism studies. Secondly, given the observational nature of our study, causal relationships cannot be definitively established, as is customary with observational studies. It remains challenging to eliminate the possibility that our findings were influenced by unmeasured confounders. Despite our diligent consideration of potential confounding factors such as age, DM, albumin, BMI, and inflammation in our analyses, certain variables might have escaped our scrutiny. Furthermore, we emphasize the importance of future studies delving deeper into these intricacies, contributing to a more nuanced understanding of the complexities involved.

## 5. Conclusions

The results of this research show the coexistence of inflammation and vitamin D_3_ receptor bb polymorphism has the highest risk of all-cause mortality compared with inflamed BB+Bb, even adjusted for other variables in women on Continuous Ambulatory Peritoneal, Automated Peritoneal Dialysis, and Hemodialysis patients.

## Figures and Tables

**Table 1:** Baseline clinical and biochemical characteristics of the total population and VDR genotypes.

**Clinical Data:**	**Total** **n = 246**	**bb** **n = 158**	**BB+Bb** **n = 88**	***p*-Value**
Age (y)	43.2 ± 1.1	42.4 ± 1.7	43.9 ± 11.0	0.271
Time on dialysis (m)	30 (1–216)	30 (15–60)	31 (16–61)	0.950
Diabetes mellitus Yes (%)	29	19	12	0.263
CAPD/HD/APD(%)	48/23/29	29/17/17	19/6/11	0.109
Calcitriol Yes (%)	26.4	28.4	22.7	0.521
CaCO_3_ Yes (%)	85.15	85	85	0.203
BMI (kg/m^2^)	25.3 ± 4.7	24.7 ± 4.1	26.2 ± 5.6	0.021
Systolic BP (mmHg)	135.4 ± 20.9	135.2 ± 20.3	135.7 ± 22.5	0.901
Diastolic BP (mmHg)	83.9 ± 14.3	84.96 ± 13.82	82.14 ± 15.10	0.220
Amenorrhea (%)	38.6	50	52.9	0.401
BMD (g/cm^2^)	0.42 (0.35–0.50)	0.43 (0.36–0.51)	0.41 (0.34–0.45)	0.102
T-score	−1.26 ± 1.06	−1.14 ± 1.09	−1.48 ± 0.97	0.031
Z-score	−1.20 (−1.75–−0.50)	−1.1 (−1.75–−0.50)	−1.30 (−1.30–−0.90)	0.012
Fat body mass(% of)	30.19 ± 9.66	30.01 ± 9.44	30.49 ± 10.07	0.970
**Biochemical Data:**				
Ca × PO_4_	45.73 ± 17.6	46.54 ± 18.49	44.27 ± 16.11	0.327
cCa(alb) (mg/dL)	9.55 ± 1.01	8.56 ± 1.58	8.50 ± 1.66	0.759
Phosphorus (mg/dL)	5.38 ± 1.90	5.42 ± 1.92	5.30 ± 1.88	0.641
Albumin (g/dL)	3.61 ± 0.72	3.69 ± 0.72	3.50 ± 0.67	0.052
PINP (ng/mL)	321 (159–652)	309 (166–647)	337 (154–675)	0.352
β-CTx (ng/mL)	1.36 (0.16–2.4)	1.30 (0.83–2.37)	1.46 (0.72–2.41)	0.470
OPG (ng/mL)	5167 (3634–7083)	5209 (3729–7105)	4759 (3285–7083)	0.810
Osteocalcin (ng/mL)	169 (64–300)	169 (70–297)	171 (53.68–300)	0.927
iPTH (pg/mL)	124 (41–337)	116 (40–319)	149 (43–342)	0.219
Fetuin A (g/L)	0.47 ± 0.11	0.48 ± 0.11	0.46 ± 0.12	0.091
hs-CRP (mg/dL)	0.34 (0.01–5.29)	0.29 (0.12–0.69)	0.52 (0.21–1.50)	0.010

Continuous Ambulatory Peritoneal Dialysis (CAPD), Automated Peritoneal Dialysis (APD), Hemodialysis (HD), Body Mass Index (BMI), Bone Mineral Density (BMD), albumin-corrected calcium cCa (alb), total pro-collagen type1-amino-terminal propeptide (PINP), C-terminal telopeptide-β aspartic acid (β-CTx), Osteoprotegerin (OPG), Intact parathyroid hormone 1–84 (iPTH), high sensitibity C-reactive protein (hs-CRP). Values are shown in mean ± standard deviation, median (IQR: 25th–75th quartiles), and percentages. The statistical tests used were Student’s *t* test, Median test, and χ^2^ with a *p* < 0.05.

**Table 2:** Clinical and biochemical characteristics according to survival status.

Group	Survivors	Non-Survivors	*p*-Value
Patient n	179	55	
Age (years)	42 ± 11	46 ± 12	0.010
Diabetes mellitus (yes/non)	48/131	29/27	0.001
Polymorphism bb/BB+Bb (%)	64.8/35.2	65.5/34.4	0.551
Time on dialysis (months)	32 (16–69)	24 (11–45)	0.022
Calcitriol yes/no (%)	75/25	76/24	0.521
CaCO_3_ yes/no (%)	14.6/85.3	11.8/88.2	0.457
BMI (kg/m^2^)	25.22 ± 4.99	25.52 ± 3.72	0.668
BMD (g/cm^2^)	0.42 (0.35–0.50)	0.43 (0.35–0.49)	0.282
Creatinine (mg/dL)	10.17 ± 3.50	9.37 ± 3.41	0.161
cCa alb (mg/dL)	8.66± 1.59	8.60 ± 1.56	0.788
Phosphorus (mg/dL)	5.39 ± 1.79	5.20 ± 2.03	0.491
hs-CRP (mg/dL)	0.32 (0.14–0.78)	0.56 (0.23–1.52)	0.001
Albumin (g/dL)	3.78 ± 0.66	3.29 ± 0.65	0.003
iPTH (pg/mL)	154 (43–344)	86 (28–318)	0.152
PINP (ng/mL)	313 (165–652)	360 (175–677)	0.750
β-CTx (ng/mL)	1.46 (0.81–2.45)	1.23 (0.81–2.24)	0.082
OPG (ng/mL)	5710 ± 3178	7236 ± 4535	0.029

Body Mass Index (BMI), Bone Mineral Density (BMD), Albumin-corrected calcium (cCa), Intact parathyroid hormone 1–84 (iPTH), high sensitivity C reactive protein (hs-CRP), total pro-collagen type1-amino-terminal propeptide (PINP), and Osteoprotegerin (OPG). Values are shown in mean ± standard deviation, median (IQR: 25th and 75th quartiles), and percentages. The statistical tests used were Student’s *t* test, Median Test, and χ^2^ with a *p* < 0.050.

**Table 3:** Crude and adjusted all-cause mortality in females on dialysis.

	Univariate		Multivariate	
Variable	HR (95% CI)	*p*-Value	HR (95% CI)	*p*-Value
Age (years)	1.04 (1.01–1.07)	0.002	1.02 (0.98–1.06)	0.341
Vintage (months)	0.98 (0.98–1.00)	0.056	0.99 (0.98–1.00)	0.790
Diabetes mellitus (presence)	2.55 (1.50–4.40)	0.001	1.83 (0.88–3.77)	0.100
Albumin (g/dL)	0.54 (0.34–0.82)	0.005	0.67 (0.39–1.14)	0.142
iPTH (pg/mL)	1.00 (0.99–1.00)	0.322	0.99 (0.99–1.00)	0.223
OPG (per 100 ng/mL)	1.00 (1.00–1.00)	0.005	1.00 (1.00–1.00)	0.320
hs-CRP > (0.3 mg/dL)	1.40 (1.18–1.66)	0.005	1.36 (1.11–1.66)	0.003

Time on dialysis (Vintage), Intact parathyroid hormone (iPTH), Osteoprotegerin (OPG), and high sensitivity-C-reactive protein (hs-CRP).

## Data Availability

The database used in the current study is not available in a public repository, but they are available from the corresponding author on reasonable request.
